# Validation of Yoruba Version of Family Burden Interview Schedule (Y-FBIS) on Caregivers of Schizophrenia Patients

**DOI:** 10.5402/2012/165179

**Published:** 2012-06-10

**Authors:** Victor Olufolahan Lasebikan

**Affiliations:** Department of Psychiatry, College of Medicine, University of Ibadan, P.O. Box 31395 (GPO), Ibadan, Nigeria

## Abstract

*Objective*. To validate the Yoruba version of Family Burden Interview Schedule (Y-FBIS) for assessing the burden on caregivers of persons with schizophrenia. *Methods*. Three hundred and sixty-eight dyads of persons with schizophrenia and their caregivers were recruited from a psychiatric outpatient clinic. The (Y-FBIS) and the Yoruba version of the GHQ-12 (Y-GHQ-12) were applied to the caregivers. Patients' level of social functioning was assessed using the Global Assessment of Functioning scale. *Results*. All (368) caregivers were used for tests of internal consistency, 180 for interrater reliability, and another 180 for test-retest reliability. Internal consistency of the Y-FBIS was demonstrated by a significant Cronbach **α** of between 0.62 and 0.82 for each item. Concurrent validity of the Y-FBIS was illustrated by its significant positive correlation with Y-GHQ-12 (*r* = 0.633 , *P* < 0.01). Split-half reliability was 0.849. Intraclass correlation coefficient for the total score of Y-FBIS was 0.849 at 95% confidence interval. Test-retest reliability of individual scales ranged from 0.780 to 0.874 and was 0.830 for total objective scale score. Convergent validity was shown by the significant positive correlation (*r* = 0.83) between the objective burden score and subjective burden score of Y-FBIS. ROC curve area was 0.981. *Conclusion*. The Y-FBIS is a valid, reliable, and sensitive instrument for assessing the burden on caregivers of persons with schizophrenia in Nigeria.

## 1. Introduction

Pai and Kapur's Family Burden Interview Schedule (FBIS) is a standardized instrument for assessing family burden [1]. The FBIS has been used to assess burden among hospital attendees, as well as those residing in the community. This instrument has been used in different studies among caregivers of patients with schizophrenia [2, 3], intellectual disability [4], and obsessive compulsive disorder [5] thereby demonstrating the scope of mental health conditions among which the instrument is applicable for assessing caregiver's burden.

During the development of the original English version of FBIS, Pai and Kapur employed free unstructured interviews with caregivers as the first step; a group of caregivers were interviewed focusing on the various areas of burden they might have experienced due to the patients' illness; these researchers recorded verbatim details of the interview [6], and the contents were thereafter analyzed in order to categorize the experienced burden. The interview schedule was reported to have lasted about 25 minutes [7].

The FBIS has been used in different studies with satisfactory psychometric properties [3, 4]. Several studies have been conducted on caregivers' burden in Nigeria [8, 9]. A burden assessment tool in a major local language in this instance Yoruba Language with established psychometric properties is needed for evaluating burden on caregivers of patients with schizophrenia. Thus, this study set out to translate the English version of the FBIS into Yoruba Language and to validate the Yoruba version of the FBIS which was further modified. The psychometric properties of the FBIS were then assessed in a sample of local caregivers of patients with schizophrenia, with the eventual aims of using this instrument in clinical settings and helping to implement programs to address the needs of caregivers of patients with schizophrenia.

## 2. Patients and Methods

### 2.1. Patients

Patients were recruited from the Psychiatric Unit of Ring Road State Hospital, the apex hospital for all hospitals under the management of Oyo State Hospitals Management Board that is situated in Ibadan capital city of the state and is the only psychiatric unit in a general hospital in Oyo state of Nigeria with a population of over 5.5 million people [10]. This unit is the only mental health service within a general hospital setting in Oyo State of Nigeria.

This was part of a larger study “Disability profile and correlates among patients with psychosis in Ibadan.” The recruitment period was between January and December 2008. All the outpatients under the care of Ring Road State Hospital, Psychiatric Unit with a principal diagnosis of schizophrenia, and their respective caregivers constituted the sample population.

Participants provided written informed consent and Ethical approval to conduct the study that was obtained from Ethical Review Committee of the Department of Planning, Research and Statistics, Ministry of Health, Oyo State, Nigeria in December 2007.

The diagnoses of the patients were made by the psychiatrist.

All of the patients recruited met the principal diagnosis of schizophrenia according to the Structural Clinical Interview for DSM IV Axis I disorder (SCID) [11]. Patients with any additional DSM IV axis I, any axis II, or axis III diagnoses were excluded from the study. Patients with any additional DSM IV axis I, any axis II, or axis III diagnoses were excluded from the study. This was to exclude burden as a result of medical comorbidity or any other psychiatric morbidity.

### 2.2. Caregivers

A “principal caregiver” was selected and for the purpose of this study defined as “a nonprofessional person in the community who was most involved with the everyday care of the case and would be very likely to respond to any request for special assistance at any time, if such a request was made by the case” [12]. In other words, such a person is nonpaid. An additional criterion was that all the recruited caregivers must have lived exclusively with the patient for at least 1 year prior to recruitment and were not involved in the care of any other family member with any mental or chronic physical illness. They were also Yoruba Speaking Nigerians of either gender aged 18 and above and were able to understand the exercise.

### 2.3. Setting of the Interview

Face-to-face interview was arranged with each patient and the principal caregiver. Caregivers were interviewed without the presence of the patients, to facilitate free expression of their feelings. The interview took place at the special Clinic of the Ring Road State Hospital. Each interview took between 40 and 50 minutes to complete.

### 2.4. Assessments

Two bilingual psychiatrists translated the original English version of FBIS into Yoruba and then backtranslated to English. The Yoruba version was modified until the backtranslated English version was comparable with the original version. An expert panel comprising specialists psychiatrists, senior psychiatric nurses, social workers, and public health nurses with at least 3 years of experience in general adult psychiatry evaluated the content validity of the modified Y-FBIS.

The Yoruba version was further modified after expert panel evaluation and then discussed in a focus group with heterogeneous composition comprising of these 2 psychiatrists, 2 mental health nurses, a social worker, a clinical psychologist, and 4 caregivers of patients with schizophrenia for its acceptability, practicality and face validity.

A pilot test of the final Yoruba version was carried out on 40 caregivers of schizophrenia patients. Practical problems concerning the understandability of the wording of the items in the version and the acceptability of the administration were addressed. Thereafter, no further modification was carried out.

A total of five items were modified/added. For example modified A1 “Has the family spent extra money due to his illness, such as settling debts, purchasing over the counter medications, paying out of pocket, paying extra on water bill, electricity bill, buying cleaning agents and items used for cleaning, renting separate apartment for patient?” this question has taken into consideration issues such as purchasing medication out of doctor's prescription, and payment of medical bills out of pocket which peculiarly characterizes care of mentally ill within our culture.

The item “how much has been spent on other treatments such as visiting herbalists, spiritualists, priests, any other alternative practitioners?” has taken into account pathway to mental health service use in Nigeria in which the majority of mentally ill use alternative practitioners.

Item A 6 was modified as follows: “Any other planned activity put off because of the financial pressure of the patient's illness: For instance, performing a religious rite, postponing a marriage, a journey, purchasing piece of land for development, building a house, training other siblings. How far is the family affected?” this item took into consideration priorities of the culture where the study took place. The Yoruba culture lays emphasis on religious rites, extravagant marriages, and investing on land and landed properties.

Item B4 added was “Patient requesting someone to help in the area of self care and other activities of daily living?” This was so added in order to take into consideration two important domains of disability in schizophrenia which are disability in self care and other activities of daily living.

Item B5 was modified as follows: “Is any other member missing or being late for school, work, and meals, and so forth?” This is because the expert panel members felt that the word missing school may be narrowing and that being late needed to be added. 

During the interview of the caregivers, family burden over the past one month was assessed by the Y-FBIS, and the administration time of Y-FBIS was recorded. The Y-FBIS was administered by a researcher who was blind to the results of all other assessments. The Yoruba version of the General Health Questionnaire-12 (Y-GHQ-12) (11) was completed by the caregivers, along with a sociodemographic questionnaire which specifically asked if they were involved and the extent of involvement with the care of the patient.

The Y-FBIS measures both objective burden and subjective burden. Objective burden is determined using 24 items grouped under 6 categories: (A) financial burden, (B) disruption of routine family activities, (C) disruption of family leisure, (D) disruption of family interaction, (E) effect on physical health of others, and (F) effect on mental health of others. Each item of objective burden is rated on a 3-point scale (0 = no burden, 1 = moderate burden, 2 = severe burden). The total objective burden score is obtained by adding the rating for each of the 24 items that ranges from 0 to 48. A supplementary item with a question asking the caregiver whether there is any other burden on the family which the rater has not asked about, followed the 6 categories of objective burden. Subjective burden is assessed by asking one standard question (“How much would you say you have suffered owing to the patient's illness?”) and scoring the answer (0 = not at all, 1 = a little, 2 = severely).

The General Health Questionnaire (GHQ) is a self-administered instrument used for screening for psychiatric morbidity [13]. It has a good internal consistency (Cronbach alpha 0.82 to 0.93) [14]. The GHQ has been validated in Nigeria [15]. The Yoruba version of the GHQ-12 (Y-GHQ-12), which was adopted to establish the concurrent validity of Y-FBIS and to assess caregivers distress, has been validated in Nigeria [16]. The GHQ had been used by researchers to assess caregiver's distress [2, 3].

The Global Assessment of Functioning (GAF) scale is a 100-point single-item rating scale used by a clinician to determine overall functioning of a patient during a particular time (e.g., at the time of the evaluation or for at least a few months during the past year). Here, functioning is considered a composite of three major areas, social functioning, occupational functioning, and psychological functioning [17]. It is derived from the Global Assessment Scale (GAS) which has established psychometric properties. Joint reliability on the GAS and the GAF scale across several studies ranged from 0.61 to 0.91 indicating fair-to-excellent agreement [18]. The functional level of the patient over the past one month was assessed in this study with GAF.

The Positive and Negative Syndrome Scale (PANSS) [19] was used for symptom rating among patients with schizophrenia. This is a 30-item, 7-point rating instrument that has adapted 18 items from the Brief Psychiatric Rating Scale (BPRS) [20] and 12 items from the Psychopathology Rating Schedule (PRS) [21]. Each item on the PANSS is accompanied by a complete definition as well as detailed anchoring criteria for all seven rating points, which represent increasing levels of psychopathology: 1 = absent, 2 = minimal, 3 = mild, 4 = moderate, 5 = moderate severe, 6 = severe, and 7 = extreme. Scoring is performed on a separate rating form in consultation with the Rating Manual. In assigning ratings, one first refers to the item definition to determine presence of a symptom. The severity of an item, if present, is then judged by using a holistic perspective in deciding which anchoring point best characterizes the patient's functioning, whether or not all elements of the description are observed. The highest applicable rating point is always assigned, even if the patient meets criteria for lower ratings as well. Of the 30 psychiatric parameters assessed on the PANSS, seven were chosen a priori to constitute a Positive Scale, seven a Negative Scale, and the remaining 16 a General Psychopathology.

The Y-FBIS scores of the whole sample of caregivers were determined for evaluation of internal consistency. A subsample (180) determined by systematic sampling of 1 in 2 caregivers was selected for testing interrater reliability. The caregivers seen by two independent researchers. One researcher conducted the Y-FBIS while the other remained silent during the interview and did the rating at the same time. The scores rated by the two researchers were thereafter compared. In order to determine test retest reliability, another 180 caregivers were selected from the whole sample. They were interviewed twice by same researcher with a time interval of 7 to 14 days. The scores of Y-FBIS in the first interview were compared with those in the second interview.

### 2.5. Statistical Analysis

The Y-FBIS internal consistency was evaluated by Cronbach alpha coefficient, and Spearman-Brown prophecy statistic was used to determine split-half reliability. Intraclass correlation coefficient was used for estimating the interrater reliability and testretest reliability of the total score of Y-FBIS. Cohen's kappa coefficient was calculated for the interrater and testretest reliability of the individual items of Y-FBIS.

Correlation coefficients were used for the association between the scores of Y-FBIS and the scores of other scales including Y-GHQ-12, and GAF. The concurrent validity and construct validity were then estimated. Pearson product-moment correlation coefficient (two-tailed) was adopted for normally distributed and Spearman rank correlation coefficient (two-tailed) was used for variables that were not normally distributed with 95% confidence interval. Statistical Package for the Social Sciences version 15.0 was used for data analysis [22].

## 3. Results

Four hundred and eight patients with the DSM IV diagnosis of schizophrenia according to the SCID were assessed for their eligibility to enter the study. Forty of them were excluded for various reasons including absence or caregiver, multiple diagnoses, medical comorbidity, and refusal to give consent. As a result, 368 patient-caregiver dyads were recruited.

 Of the 368 patients interviewed, 154 (41.9%) were female, and 214 (58.1%) were male (Table 1). Their age ranged from 14 to 58 years, with a median of 32 years. Only 61 (16.6%) were married, 163 (44.3%) were single, 118 (32.1%) were separated, 11 (3.0%) were divorced, and 5 (1.4%) widowed.

Seventy-six (20.7%) had no formal education, while the rest, 292 (79.3%) had at least some elementary education. The median years of education were 4 years. The majority, 253 (68.8%) were unemployed. The mean age of onset of schizophrenia was 19 years (SD = 4.6), while the median was 23 years. The mean duration of illness was 3.4 years (SD = 2.7), while the median was 2 years. The mean GAF score was 54.6 (SD = 5.7), while the median was 50%.

Of the 368 caregivers interviewed, 43 (11.7%) were spouses, 300 (81.5%) were parents, the rest, 57 (6.8%) were nonparent family members. Two hundred and eighty six (77.8%) were female and 82 (46.8%) were male (Table 2). Sixty six (17.2%) were married, the rest, 302 (82.9%) were either single, separated, widowed, or divorced. Their age ranged from 18 to 82 with a mean of 58.1 years (SD = 19.6), median was 51 years.

Their mean years of education was 1.8 (SD = 0.9); 50.0% of them had no formal education, 35.5% had some elementary education, and 8.9% had at least some secondary school education while 5.5% had some post secondary education.

More than half (60.3%) of the caregivers were employed. The mean number of years living with the patient was 11.7 (SD = 7.4), and the mean duration of caregiving was 2.2 years (SD = 1.4). The average number of hours per week in contact with the patient was 73.7 (SD = 39.6).

 The total score of Y-FBIS had a significant positive correlation with Y-GHQ-12, (*r* = 0.744, *P* < 0.01). All subscales of Y-FBIS also positively correlated with Y-GHQ-12 score with correlations ranging from 0.635 to 0.784.

Internal consistency of the Y-FBIS was demonstrated by a significant Cronbach *α* of between 0.62 and 0.82 for each item. Concurrent validity of the Y-FBIS was illustrated by its significant positive correlation with Y-GHQ-12 (*r* = 0.744, *P* < 0.01) and the PANNS (*r* = 0.61, *P* < 0.01). Split half reliability was obtained by dividing the 24 items 2. Cronbach *α* for first 12 items (A1-C1) was 0.872 and 0.758 for the other half (C2-F2). Split half reliability was 0.849.

Intraclass correlation coefficient for the total score of Y-FBIS was 0.849 at 95% confidence interval. Cohen Kappa for the agreement between two raters on the individual items of Y-FBIS ranged from 0.745 to 0.920. Test retest reliability of individual scales ranged from 0.780 to 0.874 and was 0.830 for total objective scale score. Convergent validity was shown by the significant positive correlation (*r* = 0.83) between the objective burden score and subjective burden score of Y-FBIS. The total score of Y-FBIS had a significant negative correlation with the GAF score (*r* = −0.721, *P* < 0.01). Each subscale score and total objective score had positive correlation with PANNS (0.53 to 0.74) but a negative correlation with GAF (−0.834 to −0.562).

The expert panel members had 74.5% to 92.0% agreement on the content validity of the 24 items of objective burden and 97.0% agreement of total objective scale score (Table 2). The lowest agreement was for subscale B (0.745) (Table 2) which may be attributed to item B4. The expert panel members were of the opinion that the question is somehow contained in B2. This item with 4 more items was modified (Table 3).

**Figure 1:**
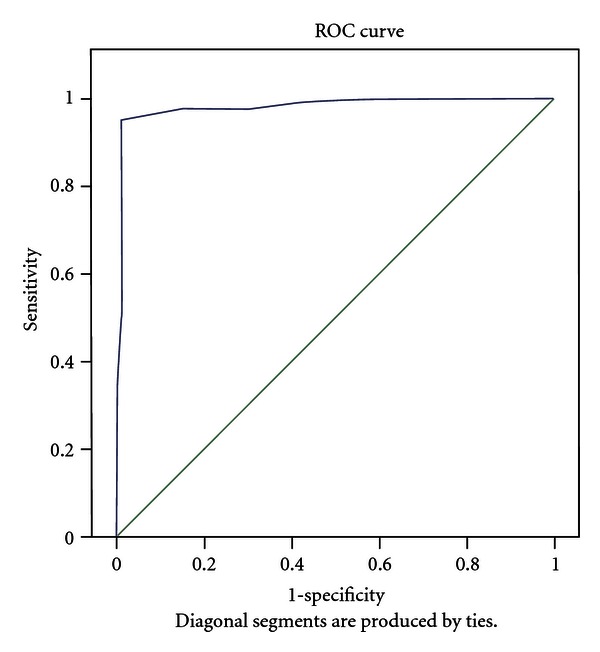
ROC Curve of YBIS and Y-GHQ-12. Area under curve = 0.981 Standard error = 0.007 95% CI = 0.967-0.995.

Figure 1 shows the ROC curve of Y-FBIS. Area under curve = 0.981, Standard error = 0.007, 95% CI = 0.967–0.995, *P* = 0.0001.

## 4. Discussion

Some of the clinical and demographic features of the sample were quite remarkable. The majority of the patients were young with short duration of illness. It is possible that reported data on age and duration of illness were mere estimates considering the widespread low level of education among the majority of both the patients and caregivers. It is also possible for respondents not to be able to accurately date onset of psychosis because of initial prodromal symptoms often somatic in nature mistaken for physical illnesses [23]. The low level of education among both study groups could be adduced to a major reason; the study center was a general hospital whose major clients were observed to be of very low socioeconomic status.

This study also noted that although more than two-thirds of the caregivers were either parent, less than a fifth of them were legally married. This is a reflection of how common mere cohabiting and procurement is in this environment [24]. The occupational status of the respondents was such that the majority of the caregivers were unskilled workers. A potential explanation is that it is more likely for an unskilled or unemployed caregiver to have time to care for his patient.

The findings for the psychometric properties of the Yoruba version of the Family Burden Interview Schedule Y-FBIS established its potential as a research instrument for measuring caregiver's burden among Yoruba speaking Nigerian caregivers of patients with schizophrenia. This involved standardized procedures including translation, and testing of the schedule in the sample. The results are discussed herein. The face validity and content validity of Y-FBIS were established by focus group discussion and expert panel evaluation. The content validity of Y-FIBS was further supported by the replies of the 368 caregivers to the questions on objective burden. Pilot test of the Y-FIBS indicated that it had acceptable administration time and that caregivers had no problem in understanding the wording of the items within the schedule.

These results demonstrated high levels of equivalence with the original English version and also to some other validated versions in some other parts of the world [1–3]. Internal consistency of the Y-FBIS was demonstrated by a significant Cronbach *α* of between 0.62 and 0.82 for each item, 0.86 for total score. These figures seem lower than 0.78–0.88 categories coefficient and 0.87 total objective Cronbach alpha reported for the validity and reliability of a Chinese version of the family burden interview schedule [3]. The present study also reported an intraclass correlation that ranged between 0.751 and 0. 988, this range is wider than 0.80–0.89 reported during validation of the Chinese version of the FBIS among caregivers of schizophrenia patients [3]. However, the 0.862 intraclass coefficient reported for total score in this study is close to the 0.87 reported in China [3]. The reliability measures obtained in this study could be regarded as substantial [25].

The convergent validity was shown by a significant positive correlation (*r* = 0.83) between the objective burden score and subjective burden score as in the studies of Pai and Kappur (*r* = 0.78) [1]. This may be surprising as a wide variation is expected between objective and subjective burden. However, a significant correlation was reported between subjective scale and Y-GHQ12 which is to be expected as the GHQ is expected to give an index of caregivers' distress.

Except for category E (effect on physical health of others), testretest reliability estimated using intraclass correlation coefficient, which controls the effect of chance, showed results that were not entirely at variance with those obtained with spearman Rho correlations. This result may indicate that sample size was adequate, and measurement errors were minimized.

 The results of the present study can be considered as good indications of the Y-FBIS's internal consistency. This is strengthened by its high specificity as demonstrated by the large area under the ROC curve. The farther away from the diagonal instrument is, the better it is from discriminating between “cases” and “non cases” demonstrating the high sensitivity of Y-FBIS. This may be attributed to the extensive and detailed process of adaptation of the scale during pilot study and the inclusion of examples in the items to assure better understanding by respondents. This can explain the high consistency of responses obtained for most subscales and the high specificity of the whole instrument. However, differences in caregivers' life conditions may contribute to different results in different studies. Usually adaptation of instruments is often a difficult task in view of cultural differences [26]; this was not so in the present study perhaps because the FBIS originates from a developing country that share certain characteristics with Nigeria.

Since there was no gold standard for Y-FBIS, concurrent validity was illustrated by its significant positive correlation with Y-GHQ-12 which is to be expected as caregivers burden is expected to be directly proportion to the presence of psychopathology and also with the Positive and Negative Symptoms Scale (PANNS) which is a measure of severity of psychosis. The YFBIS was negatively correlated with GAF suggesting that caregivers burden reduces as psychological, occupational, and social functioning increases. A weakness in this study is the inability to test for concurrent validity using another burden scale.

The expert panel members' perfect level of agreement [27] on the content validity of the 24 items of objective burden further strengthens the reliability of the Y-FBIS.

 The modified items on the Y-FBIS were based on results of focus group discussion, specifically item A3 took into consideration issues such as purchasing medication out of doctor's prescription, and payment of medical bills out of pocket which peculiarly characterizes care of mentally ill within our culture [28]. That item also took into consideration pathway to mental health service use in Nigeria in which the majority of mentally ill use alternative practitioners as first choice of service and a good proportion combine their use with current formal mental health service [24].

Y-FBIS can give information on the extent of burden on caregivers of schizophrenic patients. Nevertheless, it does not provide any cut-off scores that can be used to detect individual family members at risk from burden. Further study might be useful to identify clinical norms or cut-off points for Y-FBIS to serve as a basis for clinical intervention for the family member most in need of help. Moreover, the response of Y-FBIS is scored on a 3-point scale (no burden, moderate burden, severe burden) and perhaps by increasing the number of anchor points for the response of Y-FBIS, a finer picture of the extent of burden on caregivers can be obtained.

##  Conflict of Interests

The authors declare that they have no conflict of interests.

## Figures and Tables

**Table 1:** Modified questions within the items of the Yoruba version of the family burden interview.

Original question	Modified/added question
Item A.3: Has he spent or lost money irrationally due to his illness?	Modified: Has the family spent extra money due to his illness, such as settling debts, purchasing over the counter medications, paying out of pocket, paying extra on water bill, electricity bill, buying cleaning agents and items used for cleaning, renting separate apartment for patient?

How much has been spent on other treatments such as temples and native healers	How much has been spent on other treatments such as visiting herbalists, spiritualists, priests, and any other alternative practitioners

Item A.6: Any other planned activity put off because of the financial pressure of the patient's illness: (for instance, postponing a marriage, a journey or a religious rite. How far is the family affected?)	Modified: Any other planned activity put off because of the financial pressure of the patient's illness. (For instance, performing a religious rite, postponing a marriage, a journey, purchasing piece of land for development, building a house, training other siblings. How far is the family affected?)

Item B.4:	Added: Patient requesting someone to help in the area of self care and other activities of daily living?

Item B.5: Is any other member missing school, meals, and so forth.?	Modified: Is any other member missing or being late for school, work, and meals, and so forth?

**Table 2:** Sociodemographic characteristics of patients and caregivers.

Sociodemographic characteristics	Patients frequency	%	Caregivers frequency	%
Age group (years)				
<25	145	39.4	8	2.2
25–34	145	39.4	36	9.8
35–44	61	16.6	88	23.9
>44	17	4.6	122	33.1

Years of education				
Nil	76	20.6	180	48.9
1–6	181	49.2	128	34.8
7–12	90	24.5	32	8.7
>12	21	5.7	28	7.6

Gender				
Male	214	58.1	80	21.7
Female	154	41.9	288	78.3

Marital status				
Married	61	16.6	62	16.8
Single	163	44.3	64	17.4
Separated	118	32.1	172	46.7
Divorced	11	3.0	40	10.9
Widowed	5	1.4	30	8.2

Occupation**				
High level professional	2	1.1	—	—
Skilled worker	24	6.5	9	2.4
Semiskilled worker	44	11.1	12	3.3
Unskilled worker	45	11.1	210	57.1
Unemployed	253	68.8	137	37.2

Relationship of caregivers to patient				
Either parent	—	—	251	68.2
Spouse	—	—	43	11.7
Sibling	—	—	49	13.3
Distant family member	—	—	30	8.2
Nonrelations	—	—	27	7.3

Mean duration of illness/care	3.2 ± 1.4	—	3.4 ± 1.7	—
Mean duration of contact with patient	—	—	69.3 ± 31.5	—

**Table 3:** Cronbach *α*, intraclass correlation, Spearman correlation, and item correlation of the Yoruba version of the family burden interview schedule.

	No. of Items	Cronbach *α* coefficient	Correlation Y-GHQ-12	Correlation GAF	Correlation PANNS	Subjective scale convergent validity	Spearman brown intraclass correlation	Interrater reliability	Testretest reliability	Kappa value
A: Financial burden	6	0.74	0.713**	− 0.562*	0.54*	—	0.745**	0.964**	0.810**	0.855**

B: Disruption of routine family activities	5	0.69	0.716**	−0.69**	0.54*	—	0.688**	0.981**	0.780**	0.745**

C: Disruption of family leisure	4	0.82	0.671**	−0.72**	0.71**	—	0.794**	0.967**	0.815**	0.920**

D: Disruption of family interaction	5	0.81	0.784**	−0.71**	0.69**	—	0.788**	0.977**	0.853**	0.910**

E: Effect on physical health of others	2	0.77	0.635*	−0.67**	0.53*	—	0.620*	0.981**	0.874**	0.875**

F: Effect on mental health of others	2	0.62	0.738**	−0.834**	0.74**	—	0.575*	0.988**	0.847**	0.825**

Total objective scale	24	0.86	0.744**	−0.721**	0.61*	0.83**	0.849**	0.987**	0.830**	0.970**

Subjective scale			0.63*							

	Split		Half	Measures						

	Cronbach *α* Coefficient A1-C1	Cronbach *α* coefficient C2-F2	Guttmann coefficient	Spearman Brown coefficient	Intraclass Correlation					
	0.872	0.758	0.506	0.522	0.849**					

**P < 0.001.

*P < 0.01.
